# Spontaneous Generation

**Published:** 1867-08

**Authors:** 


### ARTICLE VII.

### *Spontaneous Generation.*

The question of spontaneous generation is one which has exercised the thoughts and pens of observers of nature from very early times. While one class of thinkers believed it possible for common inorganic matter, under certain circumstances of heat, moisture, &c., spontaneously to assume living organic forms of plants or animals, others clung tenaciously to the dictum, *omnia ab ovo*, and insisted that a préexisting living germ was essential to all living being. Aristotle believed that whenever a moist substance became dry, or a dry substance moist, animals were produced, and we find some of the poets accounting for the renovation of animal life after the deluge of Deuscaleon, by the joint action of heat and moisture upon the prolific earth.

It is undeniable that our experience among the more fully developed animals and plants, would lead us to maintain the doctrine enunciated in the old Latin maxim we have just quoted, but when we come to those low organisms which hover upon the borders of vitality, we meet with difficulties not easily overcome. It is useless to speculate upon the ridiculous blunder of Mr. Crosse, of which so much use was made by the ingenious author of the Vestiges of Creation. Those of our readers who have perused that specious and shallow book, will remember that Mr. Crosse imagined that he had made acari by passing a current of electricity through a solution of silicate of potash containing a stone. Examination, however, showed that the electrical spider was simply the common *Acarus domesticus* a common pest of all dusty localities.

There are much lower forms than this, which are continually generated in infusions or solutions of vegetable or animal matter exposed to the air, over the true origin of which a discussion is still going on. Among the most common of these is our ordinary mould, which is in truth as well defined a vegetable organism as the mushroom. The yeast plant is another of these microscopic plants, which invariably makes its appearance when sugar is passing into alcohol. After the alcohol has been formed, and vinegar is in process of formation, another mycoderm, the vinegar plant or mother of vinegar makes its appearance. In the infusions already alluded to, multitudes of vegetable and animal organisms are speedily formed.

It is to such rapid developements of life as these that the advocates of the doctrine of spontaneous generation are in the habit of appealing. They maintain that when all sources of error are avoided, these minute creatures still appear, while their opponents insist that something has been overlooked, and that these forms are regularly hatched from germs floating about in the atmosphere in the shape of impalpable dust. Several experiments had already shown that the existence of such spores was highly probable, but it was reserved for M. Pasteur, a chemist of note, to make the most conclusive researches upon this subject, which have yet been undertaken.

The first point was to prove the existence of these germs. To accomplish this, M. Pasteur drew a volume of air through a glass tube containing a plug of gun-cotton, which intercepted the particles of dust. Transferred to ether, that solvent completely dissolved the gun-cotton, and left the dust unattacked. This soon settled to the bottom, when it was removed and subjected to microscopic examination. It was found to consist of various abraded fragments of clothing, fuel, &c., and also of minute organized corpuscles, apparently spores of fungi and eggs of animalculæ. To prove their true character an experiment was undertaken. A mixture of a solution of sugar with yeast was boiled in a glass flask communicating with the air by means of a tube of platinum. In this way the air was driven out of the flask, and before it could return, the platinum tube was heated to redness. Thus all the germs were destroyed, and the air that entered was entirely free from living organisms. The flask was then hermetically sealed by fusing the glass neck. It was found that in this condition the solution might be indefinitely kept without any developement of vegetable or animal life.

If into such a flask a little of this dust already mentioned is introduced, and the flask be then hermetically sealed, the ordinary living forms are soon found. Attempts were made to collect air for these experiments over mercury, but they always failed, because this metal could not be freed from atmospheric dust containing germs.

Researches into the distribution of these germs showed that they were more abundant upon plains than upon high mountains. In all cases, air free from these germs cannot promote fermentation.

These experiments were very ingeniously varied and repeated a great number of times, so as completely to justify the conclusion of M. Pasteur, that the theory respecting the spontaneous formation of the lowest type of life is deprived of one of its essential foundations.

Donnè, however, has recently (August, 1866,) deserted the ranks of Pasteur and gone over to the advocates of spontaneous generation. He examined rotten eggs, and found that they contained no organisms. He made a hole in each with a red hot needle, and let part of the contents flow out, and then put the eggs into a pan of boiling water. Ten days afterwards, the drops of water taken from the surface contained no trace of living being, but the interior of the eggs were full of them. Donnè asks whence the germs could have come through the boiling water.

Pasteur replies that the temperature of ebullition does not necessarily annihilate the latent life of a germ, and cites instances of seeds preserving vitality after four hours boiling. Donnè in rejoinder, varies the experiment by letting out a third of the contents of the eggs as before, and instantly admitting boiling water, after which he seals the opening with wax completely closing all communication with the air.

An English observer, Dr. Child, has repeated Pasteur's experiments, but in spite of every "exaggerated" precaution finds organisms in eight out of thirteen experiments. He accounts for this discrepancy by supposing that the microscopic power used by Pasteur (350 diameter,) was not high enough. He says that it requires a power of 1500 or 1700 diameters for the satisfactory investigation of these minute objects.

Thus the matter stands at present. The analogies of generation in the higher animals and plants are opposed to the theory of spontaneous generation, but no *a priori* reasoning can settle the question. The careful observations of facts alone can decide it.

---

